# Nanomaterial-decorated micromotors for enhanced photoacoustic imaging

**DOI:** 10.1007/s12213-023-00156-7

**Published:** 2023-04-22

**Authors:** Azaam Aziz, Richard Nauber, Ana Sánchez Iglesias, Min Tang, Libo Ma, Luis M. Liz-Marzán, Oliver G. Schmidt, Mariana Medina-Sánchez

**Affiliations:** 1 Micro- and NanoBiomedical Engineering Group (MNBE), Institute for Integrative Nanosciences, Leibniz Institute for Solid State and Materials Research, Helmholtzstraße 20, 01069 Dresden, Saxony Germany; 2 CIC biomaGUNE, Basque Research and Technology Alliance (BRTA), Paseo de Miramon 182, 20014 Donostia-San Sebastián, Spain; 3 Biomedical Research Networking Center for Bioengineering, Biomaterials and Nanomedicine, CIBER-BBN, 20014 Donostia-San Sebastián, Spain; 4 Ikerbasque, Basque Foundation for Science, 48009 Bilbao, Spain; 5 Center for Materials, Architectures and Integration of Nanomembranes (MAIN), TU Chemnitz, Reichenhainer Strasse 10, 09107 Chemnitz, Saxony Germany; 6 School of Science, TU Dresden, 01062 Dresden, Saxony Germany; 7 Chair of Micro- and NanoSystems, Center for Molecular Bioengineering (B CUBE), Dresden University of Technology, Tatzberg 41, 01062 Dresden, Germany

**Keywords:** Photoacoustics, Micromotors, Closed-loop control, Gold nanorods/stars, Medical imaging

## Abstract

Micro-and nanorobots have the potential to perform non-invasive drug delivery, sensing, and surgery in living organisms, with the aid of diverse medical imaging techniques. To perform such actions, microrobots require high spatiotemporal resolution tracking with real-time closed-loop feedback. To that end,  photoacoustic imaging has appeared as a promising technique for imaging microrobots in deep tissue with higher molecular specificity and contrast. Here, we present different strategies to track magnetically-driven micromotors with improved contrast and specificity using dedicated contrast agents (Au nanorods and nanostars). Furthermore, we discuss the possibility of improving the light absorption properties of the employed nanomaterials considering possible light scattering and coupling to the underlying metal-oxide layers on the micromotor’s surface. For that, 2D COMSOL simulation and experimental results were correlated, confirming that an increased spacing between the Au-nanostructures and the increase of thickness of the underlying oxide layer lead to enhanced light absorption and preservation of the characteristic absorption peak. These characteristics are important when visualizing the micromotors in a complex in vivo environment, to distinguish them from the light absorption properties of the surrounding natural chromophores.

**Supplementary Information:**

The online version contains supplementary material available at 10.1007/s12213-023-00156-7.

## Introduction

Micromotors can maneuver through the body to perform assigned medical tasks as they possess the capacity of reaching hard-to-access locations [1–3]. Micromotor-assisted targeted drug delivery [4–7], biopsy [8], blood clot removal [9], or cell transport [10] have shown promising results. However, there are still significant limitations when steering micromotors in living organisms [2, 11], in particular when the intended application and micromotor type require high spatiotemporal resolution with precise anatomical positioning. Medical imaging or tracking of such robots in vivo is crucial for achieving effective control in a complex environment. Researchers have implemented numerous imaging techniques for microrobot monitoring, such as ultrasound (US), magnetic resonance imaging (MRI), positron emission tomography-computed tomography (PET-CT), and single-photon emission computed tomography (SPECT) [12–16]. US offers high penetration depth but low signal-to-noise ratio and spatial resolution. US Doppler and phase analysis techniques can further improve the contrast and spatial resolution in an echogenic and dynamic environment [17]. MRI has better imaging contrast for soft tissues than other conventional techniques, but its spatiotemporal resolution is insufficient to visualize small robots. CT provides deep tissue penetration but poor temporal resolution and long-term exposure might harm living tissue. PET and SPECT provide high sensitivity and molecular information, but the radiation dose remains the foremost concern. Optical methods including fluorescence [18], reflection-based IR imaging [19], or optical coherence tomography (OCT) [20], have been used to track microrobots below scattering tissues with good spatiotemporal resolution but with limited penetration depth [21, 22]. Spatial resolution degrades significantly with depth for optical methods due to pronounced light scattering by tissue.

Photoacoustic imaging (PAI) combines the high spatial resolution of optics and the imaging depth of US in tissue. The use of PAI to track micromotors was first suggested by a part of us in 2017 [2]. At that time, we visualized in real-time single magnetically-driven micromotors (up to 100 µm long) in 3D, below 1 cm thick chicken tissue [23–25]. Later, PAI has been employed for guiding capsules containing catalytic micromotors in mice intestines [26], for monitoring swarms of magnetic spiral-like micromotors to treat induced subcutaneous bacterial infection [27], and multifunctional urease-based therapeutic nanorobots inside the bladder of the mouse [28]. The tracking of cell-sized magnetic microparticles circulating in the mouse brain towards intravascular applications has also been reported [29]. Different from tissue and body fluids, microrobots are often covered with absorbing metal layers, which produce an enhanced PA signal for tracking. A suitable contrast agent can thus further enhance the PA signal for the intended application in deep tissue where the PA signal degrades with depth. Actuation and closed-loop control are other prerequisites to maneuver the microrobot in deep tissue with the required precision to target the disease region.

Here, we present the functionalization of Janus magnetic micromotors with gold nanorods (AuNRs) and gold nanostars (AuNSs) to enhance the PA contrast. Such Au nanoparticles have a better capacity to absorb light and provide thermoelastic expansion which features a superior PA response as compared to other nanomaterials. We also present the motion characteristics of micromotors in a parametric study using optical feedback control. Additionally, we show that by preventing plasmon coupling between Au-nanoparticles and the underlying metal layer by increasing the thickness of the oxide layer in between them, the characteristic absorption peak of the employed Au nanomaterials was preserved and the contrast was further enhanced, which was also validated by 2D COMSOL simulations. 

## Experimental results

### Fabrication of micromotors and characterization of the motion behavior

**Fig. 1:**
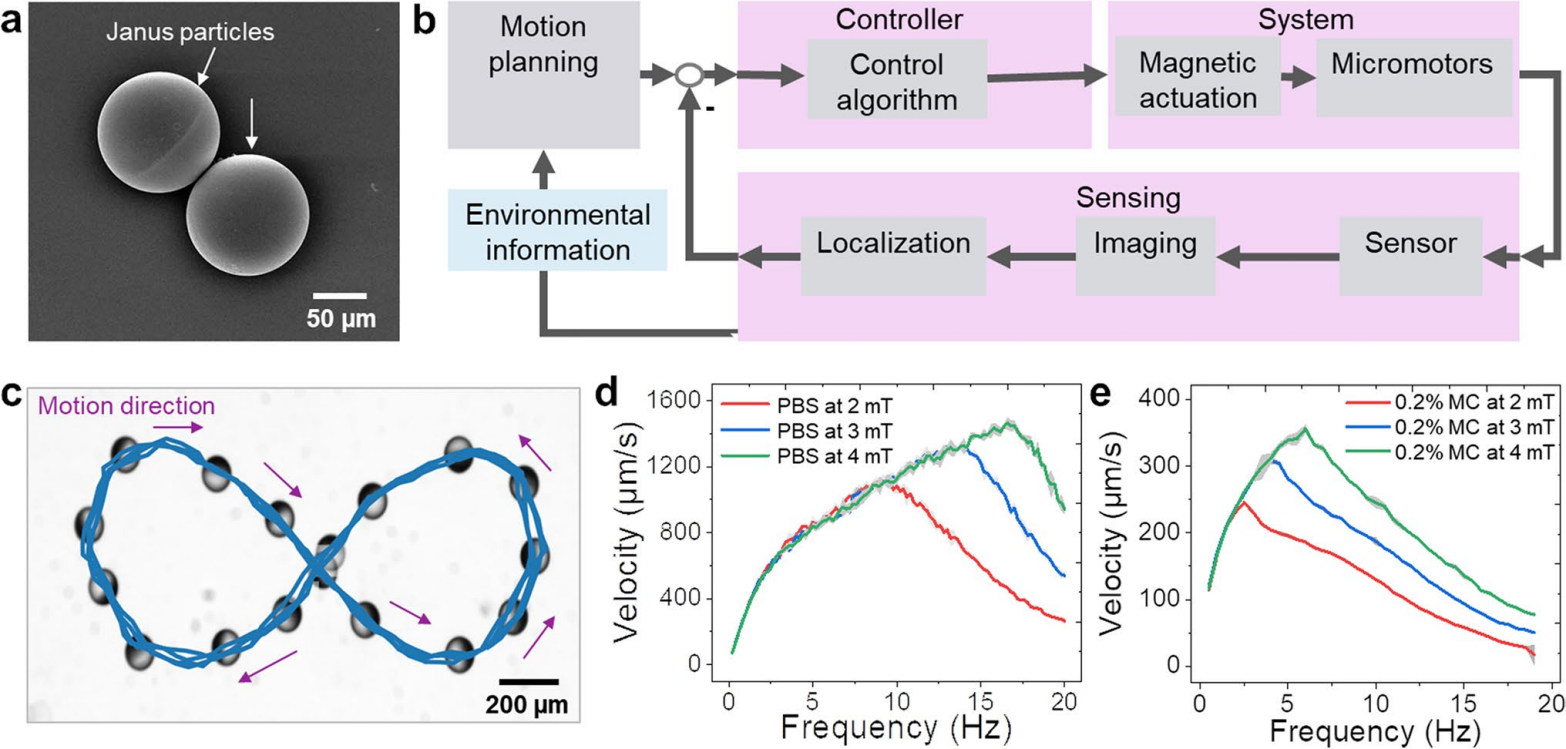
Fabrication and characterization of the motion behavior; **a**) SEM image of a metal-coated Janus particle for the tracking experiments. **b**) Block diagram showing the closed-loop control optical planning. **c**) A single micromotor following a figure-of-8 shape through closed-loop control. The arrows show the direction of motion. **d**,**e**) Parametric study of the translational speed to the rotational frequency (from 0.5 Hz to 20 Hz in 0.5 Hz steps) and the strength (2, 3 and 4 mT) of the magnetic field in PBS (**d**) and 0.2% Methylcellulose (MC) (**e**)

The micromotors were fabricated using drop-casting followed by thin metal layer deposition. First, glass slides (15 × 15 mm^2^) were sonicated in acetone and isopropanol for 3 min each and subsequently dried with an N_2_ gun. The substrates were further plasma-treated to remove impurities and contaminants and to obtain clean and hydrophilic glass surfaces. A monolayer of silicon dioxide (SiO_2_) particles (⌀ = 100 µm) was then assembled. Briefly, SiO_2_ particles were washed threefold with methanol, centrifuged for 1 min to remove the supernatant, and suspended again before usage. Silica particles were mixed thoroughly in methanol and ~ 15 µL of the particle-solvent dispersion was drop-casted on the edge of the cleaned glass slide in a tilted angle, to achieve a homogeneous layer. Microarrays were randomly formed in the direction of solvent evaporation. The resulting monolayer was dried in the air at room temperature. Finally, the samples were half-coated with Ti (10 nm), Fe (50 nm), and Ti (10 nm) by using electron beam physical vapor deposition, at a deposition rate between 0.5–1.0 Å/s. Scanning electron microscopy (SEM) was performed after coating the sample with ~ 10 nm Pt to make the specimen conductive and to avoid charging effects during imaging. Figure 1a shows an SEM image of a metal-coated Janus particle before the deposition of Au nanoparticles. Afterward, the microstructures were coated with SiO_2_ to facilitate surface functionalization with Au-nanoparticles.

The motion behavior of the micromotors was characterized through a parametric study using closed-loop control with optical feedback and real-time magnetic actuation (Fig. 1b**)**. The micromotors were immersed in DI water inside an enclosed channel and then actuated and steered by an external rotating magnetic field. The interaction of the Fe layer with the magnetic field results in torque and a respective change of orientation of the micromotor. Upon application of a rotating magnetic field, it will rotate and translate in the direction perpendicular to the rotational axis through a rolling-like interaction with the surface [30]. The steering of micromotors is realized based on real-time imaging feedback: An inverted microscope (Eclipse Ti2-E, Nikon Corp., Japan) with a digital camera (a2A2590-60ucPRO, Basler AG, Germany) captures monochromatic bright-field image frames (2592 × 1944 pixels, 8 bit per pixel) at 20 Hz. Real-time image processing for binarization with a configurable threshold and calculation of the centroid was implemented based on CuPy [31] and performed on a GPU (GeForce RTX 3090, Nvidia, USA). The localized micromotors are linked between consecutive frames using TrackPy [32] running on CPUs (2 × Intel Xeon Gold 5217, Intel, USA). The difference vector to the target location was fed to the position controller, which calculates the magnetic field vector to achieve a rolling motion in that direction as shown in the time-lapse (20 × speed) of closed-loop control of a micromotor (Video S1). The magnetic field of 2 mT and varying rotational frequency was generated with a commercial 8-coil setup (MFG-100-i, Magnebotix AG, Switzerland). The motion planning determines the target location to follow a figure-of-8 shape (Fig. 1c).

For the parametric study, the average velocity over one revolution was determined for rotational frequencies of 0.5 Hz to 20 Hz in 0.5 Hz steps, with 3 repetitions. The experiments were performed in a solution of PBS and 0.2% MC, which mimic the rheological properties of a variety of biological fluids, and under varying magnetic field strength (2, 3, 4 mT) (Fig. 1d and e). In all cases, a characteristic behavior emerges for the translational speed in relation to the rotational frequency. A monotonic increase was observed until a step-out frequency was reached, followed by a sharp decline in the speed. Both the step-out-frequency and the maximum speed were found to increase with increasing magnetic field strength. The micromotor’s speed reduces by approximately a quarter in MC, due to the higher viscosity and increased drag of 0.2% MC compared to PBS.

### Synthesis of Au nanoparticles and deposition on micromotors

Gold nanostars (AuNSs) were prepared using a surfactant-free method assisted by silver ions [33]. ~ 14 nm gold seeds (1.5 mL, [Au^0^] = 0.5 mM) prepared by the Turkevich method [34] were added to an aqueous solution (200 mL) containing hydrogen tetrachloroaurate trihydrate (HAuCl_4_) (1 mL, 50 mM) and HCl (0.2 mL, 1 M), followed by a fast addition of silver nitrate (AgNO_3_) (0.6 mL, 10 mM) and ascorbic acid (AA) (1 mL, 100 mM) under vigorous stirring. After 30 s, an aqueous hexadecyltrimethylammonium bromide (CTAB) solution (4 mL, 100 mM) was added to the mixture to increase the colloidal stability of the AuNSs. Upon synthesis, the solution was centrifuged (3500 rpm, 30 min) to remove excess reactants and dispersed in CTAB solution (1 mM). The final gold concentration was 1 mM. The average diameter determined by measuring the dimensions from the transmission electron microscopy (TEM) images was 80 ± 3.

Gold nanorods (AuNRs) were prepared using Ag-assisted seeded growth [35]. Gold seeds were synthesized by fast reduction of HAuCl_4_ with sodium borohydride (NaBH_4_)in CTAB solution. HAuCl_4_ solution (0.025 mL, 50 mM) solution was added to a solution of CTAB (4.7 mL, 100 mM). Afterward, a freshly prepared NaBH_4_ (0.3 mL, 10 mM) solution was rapidly injected under vigorous stirring. The solution color changed from yellow to brownish yellow and the stirring was stopped after 2 min. The gold seed solution was aged at room temperature for 30 min before use. To prepare the growth solution, 9.0 g of CTAB and 1.234 g of NaOH were dissolved in 500 mL of warm Milli-Q water (~ 50 ºC) in a 1 L Erlenmeyer flask. Once the sodium oleate was completely dissolved, the mixture was cooled down to 30 ºC and AgNO_3_ (24 mL, 4 mM) under stirring. The mixture was kept at 30 ºC for 15 min after which HAuCl_4_ was added (2.5 mL, 100 mM) under vigorous stirring. The mixture became colorless after 20 min at 30 ºC and after the introduction of HCl (2.1 mL, 37%). After 15 min of stirring, AA (1.25 mL, 64 mM) was added, and the solution was vigorously stirred for 30 s. Finally, the seed solution (0.8 mL, 0.25 mM) was injected into the growth solution under vigorous stirring for 5 min, and then the solution was left undisturbed at 30 ºC for 12 h. The solution was centrifuged twice (8000 rpm, 30 min) to remove excess reactants and dispersed in an aqueous CTAB solution (1 mM). The final gold concentration was 0.5 mM. The average length and diameter (in nm) determined by measuring the dimensions from the TEM images were 83 ± 5 and 18 ± 1, respectively.

Mercapto poly (ethylene glycol) carboxylic acid (PEG) with a molecular weight of 10 kg/mol was used for ligand exchange [36]. An aqueous solution of PEG (5 mL) containing 50 molecules/nm^2^ was added dropwise to the solution of gold nanoparticles (50 mL, 0.5 mM) under vigorous stirring. The mixture was reacted for about 1 h. PEG-modified gold nanoparticles were centrifuged twice (previous conditions) and finally dispersed in water.

**Fig. 2:**
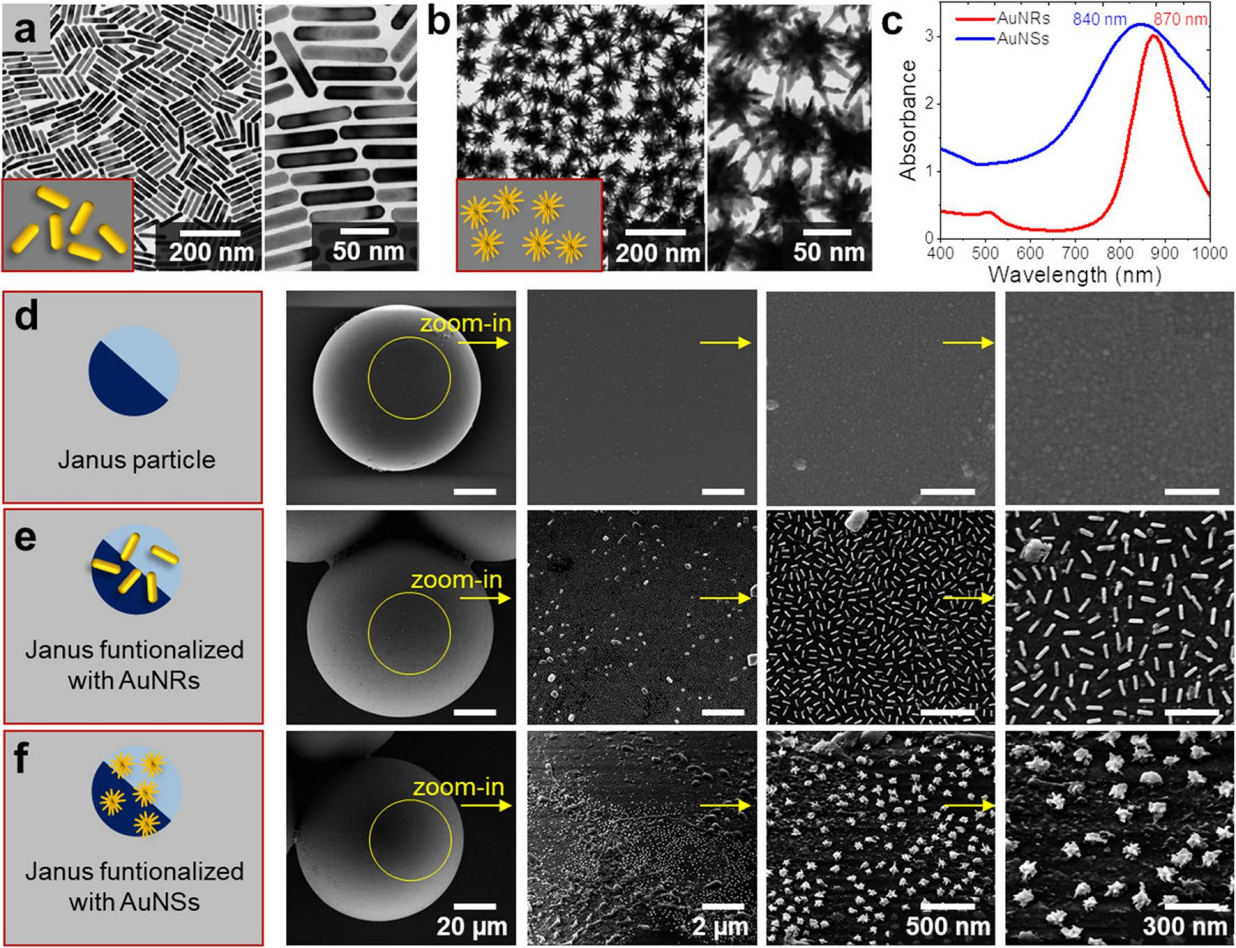
Fabrication and characterization of Au nanoparticle-coated micromotors; **a**, **b**) Representative TEM images of AuNRs (**a**) and AuNSs (**b**). **c**) UV–Vis-NIR spectra of AuNRs and AuNSs in aqueous dispersion. **d**) Sketch of a half-metal coated Janus particle and SEM images showing the surface without Au-nanoparticles. **e**, **f**) Sketches and SEM images of Janus particle surfaces functionalized with AuNRs (**e**) and AuNSs (**f**)

TEM images of AuNRs and AuNSs were obtained with a JEOL JEM-1400PLUS transmission electron microscope operating at an acceleration voltage of 120 kV using carbon-coated 400 square mesh copper grids as shown in Fig. 2a and b respectively. UV–Vis-NIR optical extinction spectra were recorded using a spectrophotometer in DI water as a medium (Fig. 2c), which featured intense localized surface plasmon resonance (LSPR) peaks at 840 nm for AuNSs and 870 nm for AuNRs.

After synthesis, carboxyl-modified AuNRs/AuNSs were immobilized on SiO_2_-coated micromotors using a previously described protocol based on (3-aminopropyl)triethoxysilane (APTES) and carbodiimide chemistry [37–39]. Briefly, oxygen plasma was applied (1 min) to activate the surface molecules of the deposited oxide, and APTES (2 wt%) solution was prepared with 5 wt% DI water and 93 wt% absolute ethanol beforehand. The micromotor sample was immersed into the APTES solution after plasma activation and incubated for 1 h at room temperature, to obtain amine groups on the micromotor surface. The samples were rinsed with absolute ethanol and phosphate-buffered saline (PBS). Then, N-ethyl-N-3-dimethylaminopropyl carbodiimide hydrochloride (EDC) at a concentration of 10 mM, and active ester compound N-hydroxy-succinimide (NHS) at a concentration of 5 mM, were prepared and used for coupling the carboxyl groups from AuNRs/AuNSs (with a concentration of 300 µg/mL) to the amino groups on the micromotor surface, forming covalent bonds. The samples were soaked for approx. 2 h at room temperature. Afterward, the samples were washed with PBS and DI water. As observed in Fig. 2d, the control sample showed no sign of Au-nanomaterial, while the functionalized samples were successfully labeled with AuNRs and AuNSs on the micromotors’ surface, respectively (Fig. 2e and f).

### Dual US and PA imaging of micromotors

Dual US and PA measurements were carried out by using the Vevo-LAZR X (FUJIFILM VisualSonics, The Netherlands) system, a multimodal platform that allows the simultaneous imaging of high-resolution US and PA. US provides anatomical and functional information while PA contributes to the molecular details. The system was equipped with a linear array US transducer at a central frequency of 21 MHz with a depth of 25 mm and fiber optic bundles on either side of the transducer for illumination. The fiber bundle was coupled to a tunable Nd: YAG laser (680 to 970 nm) with a 20 Hz repetition rate and the signals were collected by the 256-element linear array transducer (with an in-plane axial resolution of 75 µm). The pulsed laser generated a wavelength-tunable pulsed beam which was delivered by a bifurcated fiber bundle integrated with the transducer. Both US and PA signals were collected and reconstructed using onboard software. For laser spectral excitation, the PA images were acquired at a wavelength range of 680–970 nm with an increment of 5 nm over the entire scan range. All the measurements were performed in DI water.

**Fig. 3:**
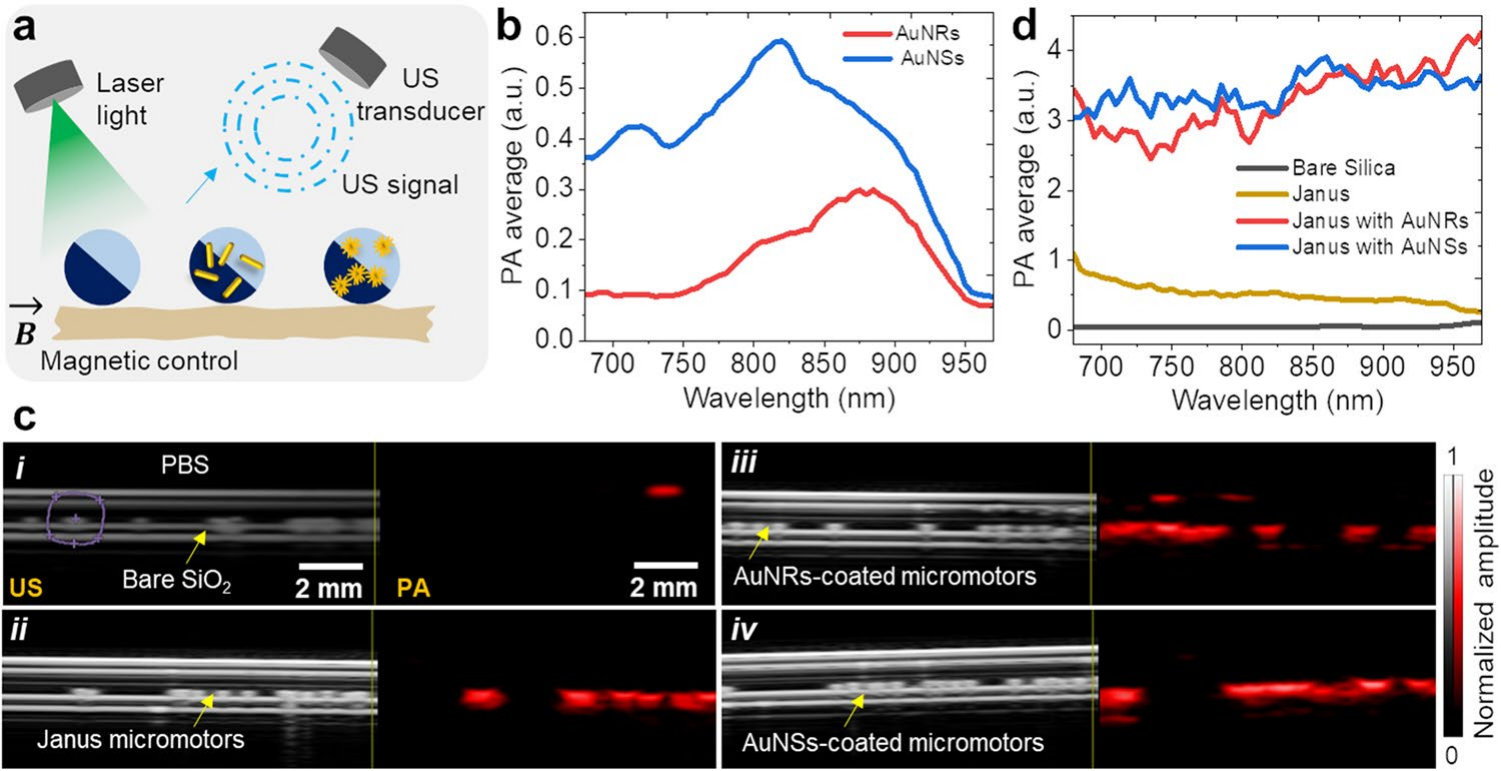
Dual US and PA imaging; **a**) Schematic of PA imaging principle with a different type of micromotors. **b**) PA spectra of AuNSs and AuNRs with absorption bands at 820 nm and 875 nm, respectively. **c**) Dual US and PA imaging of bare SiO_2_ particles (⌀ = 100 µm) (i), half-metal coated particles (Ti = 10 nm, Fe = 50 nm, Ti = 10 nm and SiO_2_ = 30 nm) (ii), AuNRs-coated (iii), and AuNSs-coated micromotors (iv). **d**) PA spectra of bare SiO_2_ particles, half-metal coated particles, AuNRs-coated, and AuNSs-coated micromotors

The imaging experiments were carried out using a dual US and PA setup, as schematically shown in Fig. 3a. A phantom setup was prepared including a water bath and enclosed tubing channel. For the tubing phantom, transparent intravascular polyurethane (IPU) tube (inner diameter ~ 380 µm, outer diameter ~ 840 µm; SAI Infusion Technologies, USA) was mounted in a water bath. The micromotors were inserted into the tube and immersed in the phantom chamber containing DI water for better acoustic coupling. The fluence was set below the Maximum Permissible Exposure (MPE) limit (20 mJ/cm^2^), followed by the safe exposure guidelines [40].

The measurements were performed with a position-fixed high-frequency transducer to avoid image distortion. PAI relies on multiwavelength excitation and subsequent spectral processing to identify optical signatures of the specific contrast agents. First of all, the NIR spectrum of AuNRs and AuNSs was recorded using PA imaging mode (Fig. 3b). Absorption bands were recorded at 820 nm for AuNSs and 875 nm for AuNRs, in agreement with the UV–Vis-NIR spectra measured by optical spectrophotometry with slight differences. The PAI system was equipped with a NIR pulsed laser and all PA images were acquired over the entire scan range of 680–970 nm, with an increment of 5 nm. Altogether, four samples were prepared in PBS as a medium, including bare SiO_2_ particles, half-metal coated particles, AuNRs-coated, and AuNSs-coated micromotors (Fig. 3c, i-iv). Dual US and PA images of all samples were captured separately and no PA signal was recorded from SiO_2_ particles, which exhibited US contrast only (Fig. 3ci). The half-metal coated particles (⌀ = 100 µm) provided both US and PA contrast because they are coated with thin absorbing adjacent metal layers (Ti = 10 nm, Fe = 50 nm, Ti = 10 nm, and SiO_2_ = 30 nm) (Fig. 3cii) [41]. Yellow arrows in the image indicate the position of a trail of micromotors, from a single to a swarm of them. The Janus micromotors inside the enclosed channel can also make small clusters in the form of dimer or trimer, which lead to enhanced PA signal intensity due to the increased IR light absorption surface. To observe the influence of particle clustering on the PA resulting signal, an additional experiment was performed by placing a single, dimer, and trimer inside the phantom tubing, and as expected there was a good agreement between the PA signal increase to the number of imaged Janus micromotors. (Fig. S1). The samples were further functionalized with AuNRs and AuNSs to enhance the PA signal contrast. Contrast agents with narrow absorption bands in the NIR are a suitable choice of exogenous contrast agents. AuNRs/AuNSs are known to be biocompatible and have successfully been implemented to improve PA contrast.

The labeled micromotors were inserted into tubing for imaging and the labeled micromotors exhibited a stronger PA signal, as compared to metal-coated micromotors (Fig. 3c, iii-iv). By plotting the recorded PA data we indeed observed an enhanced PA signal from labeled micromotors (Fig. 3d), which is crucial for deep tissue tracking applications. Although both nanoparticle samples display absorption bands around 820–875 nm, it was not possible to observe such bands upon deposition on the half-coated SiO_2_ particles. Both samples provided comparable PA signals over a broad spectral range, meaning that this approach can improve the PA signal of micromotors in hard-to-reach regions. All PA measurements were performed at a gain of 40 dB. The employed micromotors and the here-evaluated multimodal imaging setup are appealing for the supervised drug cargo-delivery towards urinary tract diseases [42], bladder cancer or infection, or towards in vivo assisted fertilization, where similar engineered parts can be used to guide or transport sperm [4, 10].

It is worth noting that non-functionalized micromotors (Janus) also absorb light but their resulting PA signals do not exhibit strong absorption signals. The reason is that the micromotors are first coated with Ti and Fe layers for further magnetic manipulation, and such layers also possess plasmon resonances but with broader absorbance spectra. For AuNRs/AuNSs-coated micromotors, there is an increase in the PA signal, as expected. However, due to the small distance between the Au-nanomaterials and the micromotor surface, plasmon coupling results in the broadening and damping of the absorbance band. This effect can be reduced by introducing a transparent layer to IR light during the synthesis of Au-nanomaterials to preserve the optical properties of Au-nanoparticles and their PA response [43].

### Effect of increased spacing between AuNRs and micromotor surface

**Fig. 4:**
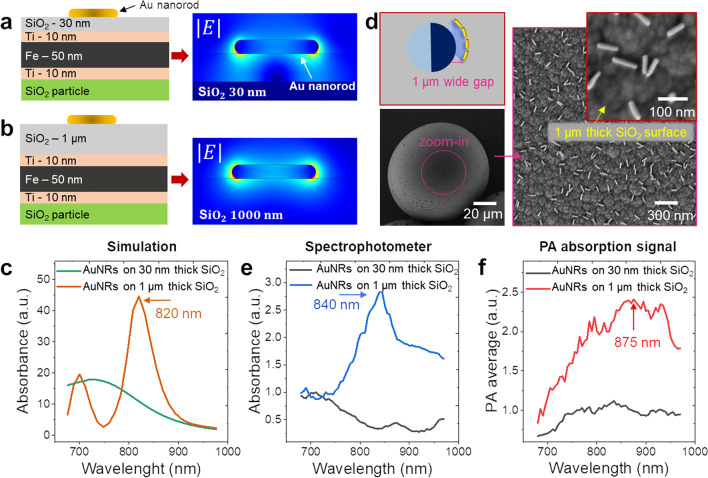
Simulation and experimental results. **a**) The layer stacks implemented were simulated using COMSOL to calculate the absorption signal for AuNRs on a metal film. **b**) An increase in absorption spectra by increasing the gap between metal and AuNRs. **c**) Simulated spectra of AuNRs (on 30 nm and 1 µm thick SiO_2_ layer) showing an enhanced absorption signal at 820 nm. **d**) Schematic representation and SEM images showing the configuration of microspheres with an insulating SiO_2_ layer on which AuNRs were deposited. **e**) Vis–NIR spectrum of AuNRs-coated micromotors with absorbance bands at 840 nm measured using a spectrophotometer. **f**) PA spectrum of AuNRs-coated micromotors with PA absorption spectra around 875 nm

To study the interaction and effect of Au-nanoparticles on the absorption signal, we implemented a 2D COMSOL simulation model, where the geometry in the out-of-plane can be regarded as uniformly distributed. We simulated AuNRs-functionalized to metal surface with varying thicknesses of transparent SiO_2_ layer (30 nm and 1 µm) as shown in Fig. 4a and b. SiO_2_ is transparent in the visible and NIR, which maintains the optical properties of AuNRs and hence their PA properties. Moreover, SiO_2_ coating has been reported to enhance the photoacoustic signal due to its higher thermal conductivity [44]. A plane TE-polarized electromagnetic wave is incident on the sample in water as a medium with a spacing distance of 30 nm between AuNRs and the metal layer. The model uses a refractive index of 1.33 for water and 1.5 interpolated wavelength-dependent refractive index for the dielectric and metal layers, which involve the COMSOL material library. The upper boundary defines the incident plane wave and the lower boundary satisfies the scattering boundary condition which absorbs the transmitted plane wave. The side boundary has Floquet conditions, meaning that the solution on one side of the geometry equals the solution on the other side multiplied by a complex-valued phase factor. This effectively turns the model into a section of a geometry that extends indefinitely in the XY plane. Initially, the AuNRs were not introduced, so the background plane wavefield was calculated. We then removed the incident wave and added a perfectly matched layer (for absorbing the incident wave to the boundary) in the four boundaries. Subsequently, AuNRs were included, and the field from the first calculation was set as the background field. We calculated the relative field from the AuNR (which can be regarded as a perturbation). If the field distribution extends to the inner side of the AuNR, it will cause more absorption. We calculated the field from 680 to 970 nm, to derive the absorption spectra, this wavelength range being identical to that in the PAI experiments. The resulting calculated field values show a strong absorption peak at 820 nm for the sample with a 1 µm spacing distance, whereas the sample with a smaller spacing distance showed a weak, broad absorption band (Fig. 4c). These results suggest that a sufficiently thick transparent layer can enhance the optical absorption signal. We, therefore, fabricated Janus microparticles with thin metal layers (Ti/Fe/Ti) and evaporated a thick layer of SiO_2_ (1000 nm) using chemical vapor deposition (CVD) before labeling them with AuNRs. SEM imaging was used to demonstrate the labeling of AuNRs on the outer surface of the new Janus samples (Fig. 4d). The recorded Vis–NIR spectrum of AuNRs coated on a 1 µm thick SiO_2_ layer shows strong absorption bands at 840 nm using the spectrophotometer (Fig. 4e). In PAI, a strong and broad absorption signal was recorded, peaking at 875 nm (Fig. 4f), in agreement with the PA spectrum of AuNRs only (see Fig. 3b). The thick silica coating reduces plasmon coupling between AuNRs and the metal layer, thereby improving the PA signal strength. The plasmon resonance band shifted up to 20 nm for the spectrophotometer study and 55 nm for PA data, as compared to the 2D simulation. This difference might be due to differences between simulation and experimental settings and the absorption band does not come from absorption only but also scattering. In PAI, it is only possible to detect absorption signatures and this might be the origin of the spectral differences. The simulation was performed with a simple 2D model to generate optical absorption signal, whereas PA takes into account different illumination and US detection mechanism. As a perspective of this work, a 3D simulation that considers different parameters such as AuNPs density, AuNPs shape, and the underlying surface geometry and composition will be realized and compared with the corresponding experimental data to evaluate their effect on the resulting PA signal strength and specificity.

## Conclusion

This work presents the functionalization of Janus micromotors with plasmonic nanomaterials, in particular AuNRs and AuNSs, which are structures with higher photothermal conversion efficiency, to enhance the PA contrast. Such labeled micromotors can provide an improved signal in deep tissue due to the presence of PA agents. As expected, AuNRs/AuNSs-coated motors exhibited enhanced PA signals as compared to bare and thin metal-coated particles. Furthermore, 2D COMSOL simulation and experimental data show that increased spacing between the AuNPs and the underlying metal layer would lead to enhanced absorption spectra which is a crucial parameter while doing imaging of micromotors in deep tissue. We also show the motion behavior of the micromotors through a parametric study using closed-loop control with optical feedback. The average velocity over one revolution is determined for rotational frequencies and a characteristic behavior emerges for the translational speed to the rotational frequency. Such feedback control algorithms can also be implemented using other medical imaging modalities to increase the future targeting efficiency of those microrobots when performing a medical operation in vivo.

### Supplementary Information

Below is the link to the electronic supplementary material.

Supplementary file1 (DOCX 91 KB)

Video S1 Time lapse (20x speed) of Janus micromotor using closed-loop control (MP4 1806 KB)

## Data Availability

The datasets generated during and/or analyzed during the current study are available from the corresponding author upon reasonable request.
