# Supplementary material for: Nanomaterial-decorated micromotors for enhanced photoacoustic imaging
Source: J Microbio Robot. 2023 Apr 22;19(1-2):37–45. doi: 10.1007/s12213-023-00156-7 (PMC10756870; doi:10.1007/s12213-023-00156-7)
Supplement: Supplementary file 1 — Supplementary file1 (DOCX 91 KB) [file 12213_2023_156_MOESM1_ESM.docx]

**Supporting Information**

**Nanomaterial-decorated micromotors for enhanced photoacoustic imaging**

*Azaam Aziz, Richard Nauber, Ana Sánchez Iglesias, Min Tang, Libo Ma, Luis M. Liz-Marzán, Oliver G. Schmidt and Mariana Medina-Sánchez**

The article includes the following supporting information;

**Video S1**. Time lapse (20x speed) of Janus micromotor using closed-loop control

**Figure S1**. PA signal intensity of single and multiple (dimer and trimer) Janus particles without Au-nanomaterial and there is an increase in PA signal by increasing the number of particles


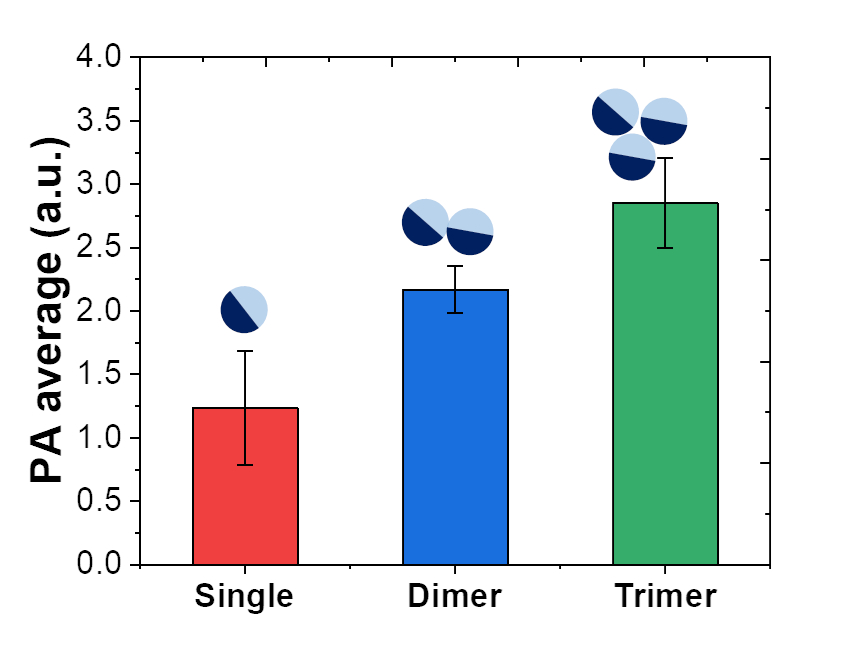


**Figure 1.** PA signal intensity of single and multiple (dimer and trimer) Janus particles without Au-nanomaterial and there is an increase in PA signal by increasing the number of particles.
